# Optimising clonal performance in sugarcane: leveraging non-additive effects via mate-allocation strategies

**DOI:** 10.3389/fpls.2023.1260517

**Published:** 2023-11-10

**Authors:** Seema Yadav, Elizabeth M. Ross, Xianming Wei, Owen Powell, Valentin Hivert, Lee T. Hickey, Felicity Atkin, Emily Deomano, Karen S. Aitken, Kai P. Voss-Fels, Ben J. Hayes

**Affiliations:** ^1^ Queensland Alliance for Agriculture and Food Science, The University of Queensland, Brisbane, QLD, Australia; ^2^ Sugar Research Australia, Mackay, QLD, Australia; ^3^ Institute for Molecular Bioscience, The University of Queensland, Brisbane, QLD, Australia; ^4^ Sugar Research Australia, Meringa Gordonvale, QLD, Australia; ^5^ Sugar Research Australia, Indooroopilly, QLD, Australia; ^6^ Agriculture and Food, Commonwealth Scientific and Industrial Research Organisation (CSIRO), Brisbane, QlD, Australia; ^7^ Department of Grapevine Breeding, Hochschule Geisenheim University, Geisenheim, Germany

**Keywords:** additive, breeding value, clonal performance, dominance, heterozygosity, non-additive effects, mate-allocation, sugarcane

## Abstract

Mate-allocation strategies in breeding programs can improve progeny performance by harnessing non-additive genetic effects. These approaches prioritise predicted progeny merit over parental breeding value, making them particularly appealing for clonally propagated crops such as sugarcane. We conducted a comparative analysis of mate-allocation strategies, exploring utilising non-additive and heterozygosity effects to maximise clonal performance with schemes that solely consider additive effects to optimise breeding value. Using phenotypic and genotypic data from a population of 2,909 clones evaluated in final assessment trials of Australian sugarcane breeding programs, we focused on three important traits: tonnes of cane per hectare (TCH), commercial cane sugar (CCS), and Fibre. By simulating families from all possible crosses (1,225) with 50 progenies each, we predicted the breeding and clonal values of progeny using two models: GBLUP (considering additive effects only) and extended-GBLUP (incorporating additive, non-additive, and heterozygosity effects). Integer linear programming was used to identify the optimal mate-allocation among selected parents. Compared to breeding value-based approaches, mate-allocation strategies based on clonal performance yielded substantial improvements, with predicted progeny values increasing by 57% for TCH, 12% for CCS, and 16% for fibre. Our simulation study highlights the effectiveness of mate-allocation approaches that exploit non-additive and heterozygosity effects, resulting in superior clonal performance. However, there was a notable decline in additive gain, particularly for TCH, likely due to significant epistatic effects. When selecting crosses based on clonal performance for TCH, the inbreeding coefficient of progeny was significantly lower compared to random mating, underscoring the advantages of leveraging non-additive and heterozygosity effects in mitigating inbreeding depression. Thus, mate-allocation strategies are recommended in clonally propagated crops to enhance clonal performance and reduce the negative impacts of inbreeding.

## 1 Introduction

Crop breeding strategies have evolved over the past two decades to meet the growing demand for food production. In particular, sugarcane breeding programs have witnessed notable advancements. These programs involve crossing between sugar-rich cultivated species, primarily *Saccharum officinarum* (2n = 8x = 80; x=10), and a wild relative, *Saccharum spontaneum* (2n = 5x-16x =40-128, x=8), which provided disease/pest resistance and abiotic tolerance in a range of varieties (Wei and Jackson, 2016; Yadav et al., 2020). Approximately 70-80% of the sugarcane genome is inherited from *S. officinarum*, while *S.spontaneum* contributes to 10-20% of the genetic makeup and the remaining 10% of the genome results from interspecific recombination (Garsmeur et al., 2018; Piperidis and D’Hont, 2020). This unique genetic configuration presents both challenges and opportunities in sugarcane breeding programs. Cultivated sugarcane varieties have a highly complex polyploid genome, exhibit a high level of heterozygosity, and can have between 100 to 130 chromosomes. A notable advantage of sugarcane lies in its ability for vegetative propagation, which facilitates fixation of traits across successive clonal generations.

More recently, the emphasis on sugarcane breeding has shifted towards crossing highly heterozygous inter-specific hybrids, aiming to generate genetic variation that can be exploited through selection in subsequent breeding cycles (Wei et al., 2021). However, the first and most challenging breeding decision is to select the appropriate genotypes as parents for crosses to maximise the performance of progeny for variety development while maintaining genetic diversity in the breeding program (Comstock et al., 1949; Boeven et al., 2020; Technow et al., 2021). The development of sugarcane cultivars typically involves extensive field testing, which takes 10 to 12 years (Park et al., 2007). In Australian breeding programs, the parental clones are primarily selected from advanced-stage trials and commercially grown cultivars, including those obtained through variety-exchange programs from overseas. The selection of parental clones is based on their additive genetic merit, considering traits such as yield, sugar and fibre content, which are predicted using best linear unbiased prediction (BLUP) approaches incorporating pedigree information (Atkin et al., 2009). Disease resistance is also a crucial factor in the evaluation process (Park et al., 2007).

Advancements in high-throughput marker technologies have facilitated the investigation of genomic prediction in sugarcane, enabling early selection for complex quantitative traits such as cane yield (Gouy et al., 2013; Deomano et al., 2020; Hayes et al., 2021; Yadav et al., 2021b). Genomic selection (GS) has the potential to expedite the breeding cycle by allowing the quick selection of superior genotypes at any stage of the sugarcane breeding program (Voss-Fels et al., 2021). In GS-assisted breeding programs, truncation selection is typically employed as the first step, where high-performing parental lines/clones are selected based on their genomic estimated breeding values (GEBVs). These selected lines/clones are then crossed randomly to produce the next generation, ensuring a high mean performance among the progeny (Wei et al., 2021). However, the mean performance of progeny can deviate from the mean breeding value of the parents due to the presence of non-additive effects.

In sugarcane breeding programs, the selection of parental clones based on GEBVs (heritable effects) should be complemented by planned mating to maximise the total clonal performance of the commercial clones. Since sugarcane is clonally propagated and highly heterozygous, both additive and non-additive genetic effects can be exploited for variety development. Mate-allocation strategies have been successfully used in animal breeding programs to manage inbreeding, preserve genetic diversity, and leverage non-additive genetic effects (Toro and Varona, 2010; Pryce et al., 2012; Aliloo et al., 2017; Gonzalez-Dieguez et al., 2019). Notably, non-additive genetic effects are substantial for a complex trait such as tonnes of cane per hectare (TCH) in sugarcane and can be captured using extended GS models (Yadav et al., 2021b). Furthermore, the presence of these non-additive effects suggests the potential for overall genetic improvement through mate-allocation strategies that specifically target dominance (for heterosis) and epistatic effects. In addition, by incorporating models that account for non-additive effects and heterozygosity, we can effectively reduce the risk of inbreeding depression in the commercial population, a concern that holds significant weight for TCH (de Azeredo et al., 2016; Silva, Gonçalves PdS, 2011). Recent studies have highlighted the importance of considering dominance effects when choosing parents based on the genomic prediction of cross-performance in clonal breeding programs utilising GS (Werner et al., 2023).

In this study, we hypothesise that mate-allocation strategies that consider non-additive effects will improve the expected performance of the clones in a sugarcane breeding program when compared to relying solely on additive genetic effects. The aim of this study is to investigate how mate-allocation strategies can enhance the prediction of clonal performance in an elite sugarcane population. First, we establish a pool of parental clones based on their GEBVs, which provide insights into the genetic potential of each parent. Subsequently, we explore two mate-allocation strategies for comparison:

- The first strategy involves exploiting only additive effects, following a traditional GBLUP model, to maximise the average breeding (additive, GEBV) value of progeny, resembling conventional mating practices.
- The second strategy leverages non-additive genetic effects and heterozygosity, in addition to the additive genetic component, through an extended-GBLUP model designed for predicting clonal performance (GPCP) to maximise clonal performance of progeny.

This study presents the results of stochastic simulations assessing the expected clonal performance and inbreeding in the next generation under these mate-allocation strategies. To determine the best mating set that optimises expected progeny merit, we utilise integer linear programming (ILP). Our analysis focuses on three commercially important traits: tonnes of cane per hectare (TCH), commercial cane sugar (CCS) for sugar content, and fibre content (Fibre).

This study’s findings hold significant implications for sugarcane breeding programs, highlighting the potential advantages of mate-allocation strategies that capitalise on both additive and non-additive genetic effects. By leveraging genomic predictions for parental selection, breeders can enhance the overall performance of sugarcane cultivars and streamline variety development processes. Moreover, incorporating non-additive effects and heterozygosity into mate-allocation decisions helps to mitigate the potential risks associated with inbreeding depression while preserving genetic diversity. These insights offer valuable guidance for future breeding strategies, fostering sustainable advancements in sugarcane productivity.

## 2 Materials and methods

### 2.1 Phenotypes and genotypes

This study used a 58K SC Affymetrix Axiom SNP array to genotype 3,006 elite sugarcane clones from Sugar Research Australia’s (SRA) breeding program (Aitken et al., 2017). The population used in this study comprised clones that were evaluated in final assessment trials (FATs) with large plots. From 2013 to 2017, the FAT series was established annually, and each series was harvested over three years (referred to as crops including the plant crop, the first ratoon, and the second ratoon crop) in Queensland’s four sugarcane growing areas: Northern (N), Burdekin (A), Central (C), and Southern (S). Within each region, there were four trials conducted each year. The clones in each FAT series were mostly repeated across at least three trials within a region, but many were unique to a specific region. In each trial, 150 to 300 clones were planted in four-row by 10-meter plots using a partially replicated design, with an average replication rate of 22%. At each harvest, three main agronomical traits, TCH, CCS, and Fibre, were measured. Data collection was focused on the middle two rows, while the outer two rows served as a buffer against competitive effects.

Before testing the genomic model, the phenotypes were adjusted for experimental and environmental effects across the series, crop, trial, and region to produce the BLUPs. It’s important to note that the BLUPs used in this study were provided by our commercial partner, SRA. SRA employed a robust general linear mixed model to address spatial variations within each trial, treating rows and columns as fixed effects within trials within regions and interaction between trials and crops with spatial variation effects, applying ASREML-R v3 (Butler. et al., 2009).


$$Y=X\;\beta +Z_{ɡ}u_{ɡ}+Z_{p}u_{p}+\varepsilon$$

Where X is the design matrix, β is the vector of fixed effects with the associated design matrix, $u_{ɡ}$ is the vector of random genotype (clone) effects for individual trials and harvests (ordered as genotypes within trials within harvests) with the associated design matrix $Z_{ɡ}$ , *u_p_* represents random peripheral effects associated with its respective designed matrix $Z_{p}$ , and the *ε* denotes the residual.

Spatial effects were accounted for by determining an optimal model within each selection trial, following the methodology outlined by Smith et al. (2007). Subsequently, based on the outcomes of this initial step, a comprehensive combined analysis over all sites was conducted. The BLUPs for the clones, based on the combined analyses, were centred for each site and then averaged over all trials and regions, which were subsequently used for genomic prediction analysis. Additional information can be obtained from the study by Wei et al. (2021) for further details on the first stage of data analysis.

A potential concern is that using BLUPs as a response variable may lead to a double penalty for the estimated genetic effects. However, in the first stage of the study, pedigree information was not incorporated, indicating that the phenotypes were not adjusted towards the pedigree before fitting the genomic prediction models. Additionally, the error variances in the FAT trials (the final step of the breeding program) were reported to be low due to the high number of replications and large plot size within the region. The degree of shrinkage in the BLUPs was rather small and consistent, given high estimates of broad-sense heritability; $H^{2}=\frac{\sigma_{ɡ}^{2}}{\sigma_{ɡ}^{2}+\frac{\sigma_{\varepsilon }^{2}\;}{r}}$ , recorded as 0.72, 0.79, and 0.89 for TCH, CCS and Fibre, respectively, across the trials. Here $\sigma_{ɡ}^{2},\sigma_{\varepsilon }^{2}$ and *r* are the genetic variance, error variance and number of replicates per clone within each trial. Furthermore, BLUPs were able to handle small amounts of missing data.

The sugarcane Axiom array contained 58,364 probe sets representing 48K single nucleotide polymorphisms (SNPs), which were highly polymorphic. All clones were screened across this array, with strict quality control measures applied. Samples that had a dish quality control (DQC) measure of less than 0.82 or a quality control (QC) call rate of less than 90% were excluded from the analysis. To ensure high-quality results, allele calling was performed using the generated cell intensity files (CEL) with Axiom Analysis Suite’s best practice workflow. Multiallelic markers were called pseudo-diploid genotypes from the array data. Aitken et al. (2016) provide detailed information on the array and genotype calling procedures. Before conducting downstream analysis, monomorphic SNP markers, as well as SNPs with a minor allele frequency (MAF) of less than 0.01, were excluded. Following quality control, the population for analysis included 2,909 clones with 26,086 highly polymorphic SNP genotypes. For each polymorphic marker, all clones (genotypes) were given a marker score of 2 if only the most frequent allele was present (i.e. homozygous for this allele), 1 if both alleles were present (i.e. heterozygous), and 0 if only the minor allele was present. All heterozygous genotypes were measured as one single-class genotype from a pseudo-diploid model during the genotype calling process (Aitken et al., 2016).

### 2.2 Simulation and genomic prediction framework

The data analysis in this study consists of two main components: one focusing on simulating the segregation of target traits in progeny and the other on predicting the breeding and clonal values of those progeny. To accurately simulate the recombination process during the formation of gametes passed from parent to progeny, it is essential to have information about marker locations on the genetic map, along with parental marker data. However, there was a challenge. Out of the high-quality 26,086 SNPs, only 4,502 were initially mapped on the available Q208 sugarcane genetic map. This presented a limitation. Despite the existence of high levels of linkage disequilibrium (LD) in sugarcane, a more extensive marker set is essential to pinpoint specific genomic regions linked to these traits (Jannoo et al., 1999; Raboin et al., 2008; Yadav et al., 2021a). To address this limitation, an LD-based algorithm was employed. This algorithm had been developed by Yadav et al. (2021a) in a prior study. Its successful application enabled us to integrate an additional 5,920 unpositioned markers onto the existing genetic map. This process resulted in a final set of 10,387 SNPs with an MAF > 0.01 on the extended genetic map (Yadav et al., 2021a). It’s important to note that these 10,387 markers, now with known positions on the map, would be used in subsequent simulations.

#### 2.2.1 Simulation of phantom progenies

**Figure 1:**
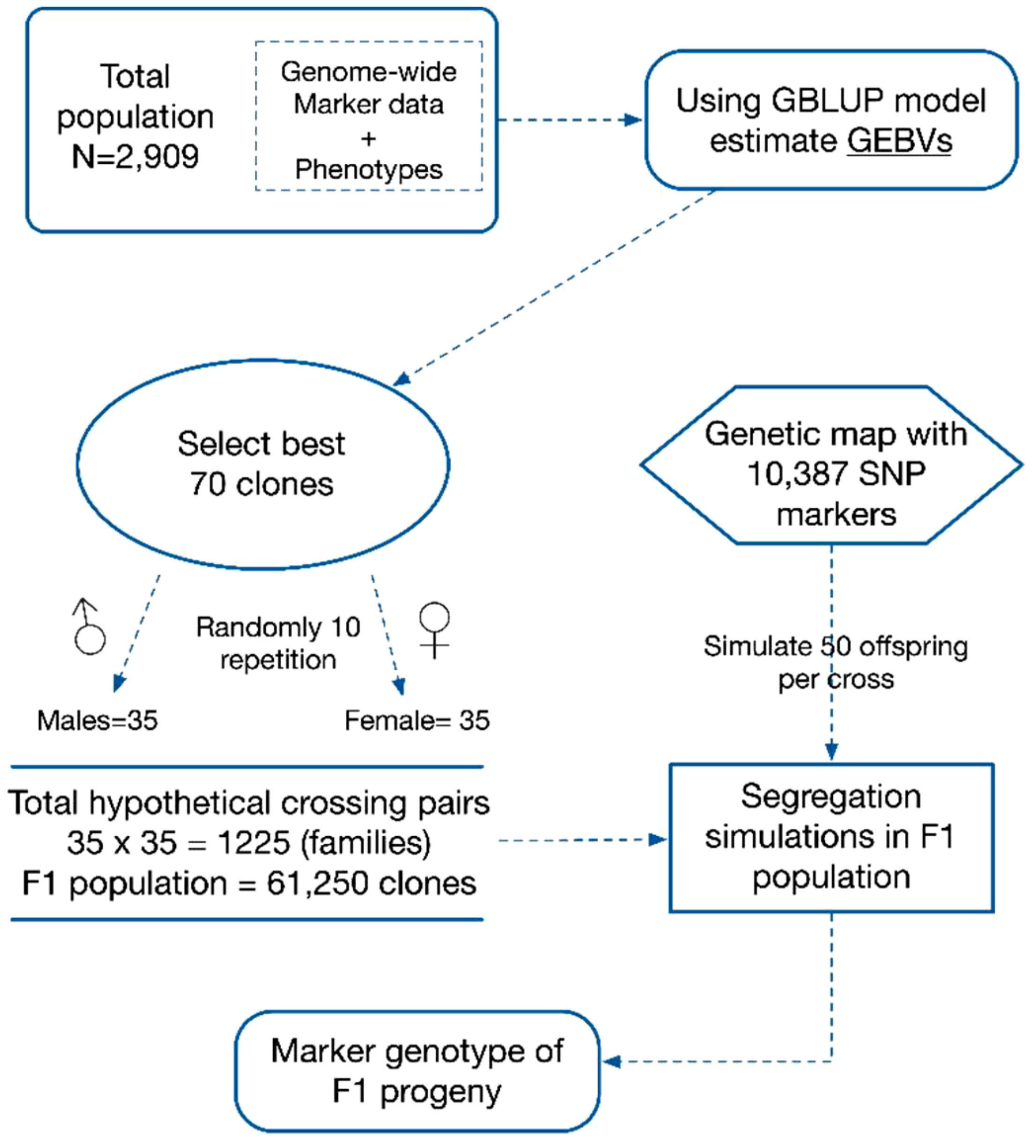
Progeny genotypes: Simulation approach illustrating the method for projecting target trait segregation patterns in phantom progenies derived from 1,225 families chosen from a pool of 2909 elite clones. The simulation process involves utilising genome-wide markers of parental clones and their corresponding positions on the linkage map. Markers with known positions, a total of 10,387, were used in the simulation.

To predict the performance of parental crosses, the first step involved simulating the genotypes of phantom progeny. In this process, 70 parents were selected from the overall population based on their GEBVs (**Figure 1**). To account for dioecy (male and female reproductive separation), 35 clones were randomly assigned as male parents, while the remaining 35 were designated as female parents from the pool of 70 parental clones. All possible crosses, totalling 1,225 (35×35) combinations, between the male and female clones were simulated. Each of these crosses produced 50 progenies. The simulation process was carried out by randomly sampling parental gametes with crossovers. This involved using an extended genetic map, having 10,387 markers with known positions (Yadav et al., 2021a).

The progenies were simulated using the R package “SelectionTools,” version 19.3 (http://population-genetics.uni-giessen.de/~software/), which utilised Plabsoft software to mimic meiosis using a count-location approach (Maurer et al., 2008). In the simulation context, the ploidy level of parental clones, which was assumed to be diploid, served as a reasonable approximation. This approach assumes that the average number of crossovers formed on a chromosome is proportional to the chromosome’s length in Morgan units. Additionally, crossover locations are distributed independently and uniformly along the chromosome. These assumptions align with the Haldane mapping function in the absence of interference (Haldane, 1919). Notably, the same software had been previously used in a recent sugarcane study to explore different approaches for implementing GS in a simulated breeding environment, considering both additive and non-additive effects for improving complex traits. Ten iterations were conducted to eliminate sampling bias between male and female parents while maintaining consistency in the selection of parental clones across iterations, enabling a comparison of the two mate-allocation strategies.

The additive genetic variance of offspring from a cross can be predicted deterministically using a combination of genome-wide marker effects, a genetic map, and phased parental haplotypes, as shown by Wolfe et al. (2021). Although we have simulated diploid inheritance, which is not appropriate for some regions of the sugarcane genome, the markers (primarily single or low dosages) in this study were chosen to have diploid-like inheritance (Aitken et al., 2016). The limitation of this approach is explored in the discussion.

#### 2.2.2 Prediction of breeding and clonal value of progeny

**Figure 2:**
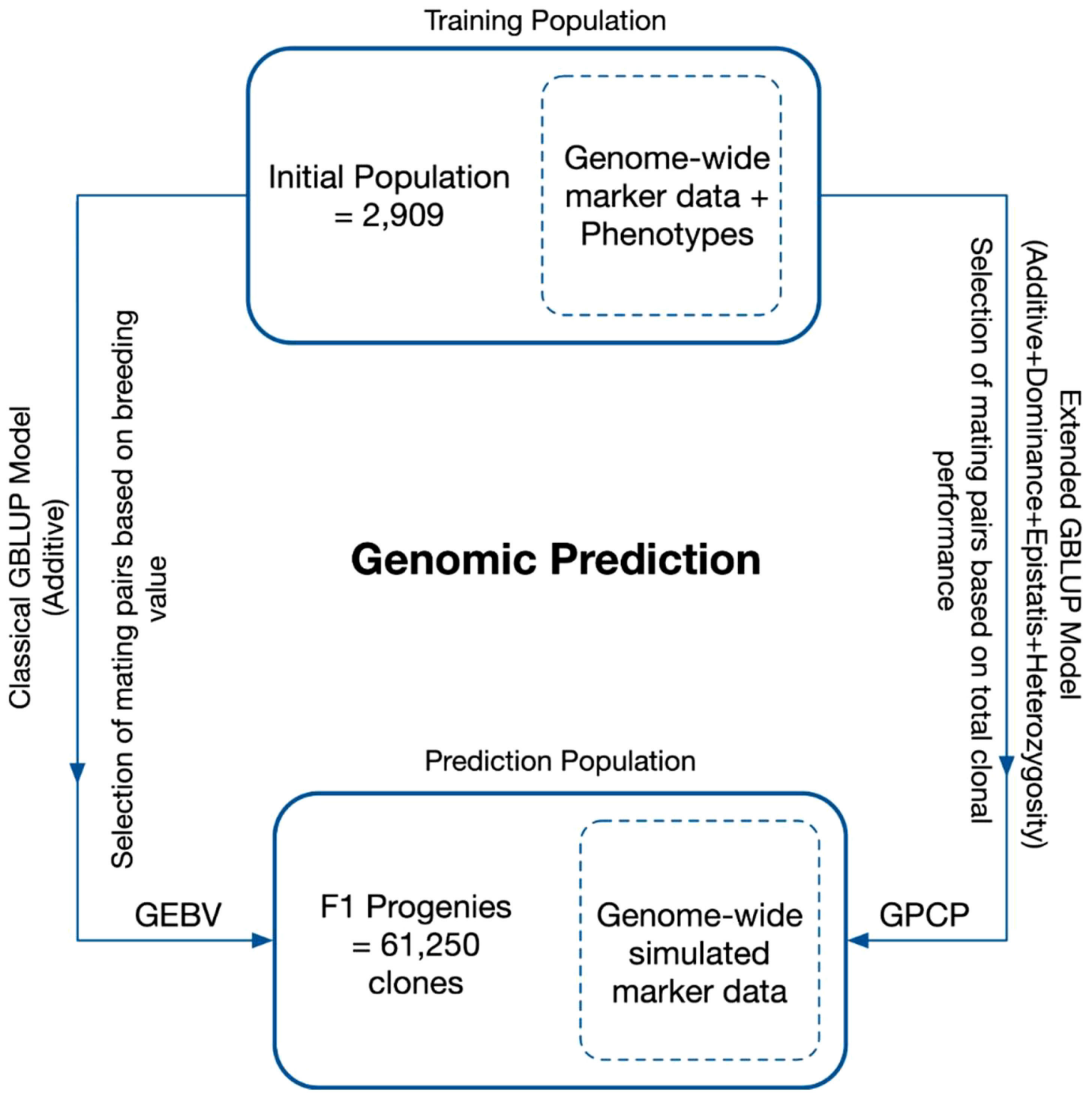
Genomic prediction framework to calculate genomic estimate breeding value (GEBV) and genomic prediction of clonal performance (GPCP) using standard GBLUP and e-GBLUP models, respectively. The models were trained using a breeding population consisting of 2,909 elite sugarcane clones with recorded phenotypes for desired traits and genome-wide marker data. The trained models were utilised to predict the performance of 61,250 simulated progenies solely based on their marker profiles.

Single-trait linear mixed models were fitted to estimate genetic variance components for TCH, CCS, and Fibre using the residual maximum likelihood (REML) approach. The variance components were estimated using initial data, which included the parental clones selected for crossing. The total population comprised 64,159 clones, with a training population consisting of 2,909 elite clones possessing both phenotypic and genotypic data. The remaining 61,250 clones (out of 64,159 clones) were simulated progenies from 1,225 families derived from the top 70 parental clones, with 50 progeny per family (**Figure 2**). The performance of these progeny in terms of breeding and clonal value was predicted using a genomic prediction framework based solely on marker profiles (**Figure 2**).

In matrix notation, the GBLUP and the extended GBLUP model can be represented as follows:


(1) $$\text{Model GBLUP }{\text{y}}^{\text{m}\times 1}=\text{Xβ}+\text{Zu}+\text{ε}$$

(2) $$\text{Model extended}–\text{GBLUP }{\text{y}}^{\text{m}\times 1}=\text{Xβ}+\text{Zu}+\text{Zd}+\text{Zt}+\text{b}\left(\text{Het}\right)+\text{ε}$$

y **^m×1^** is a vector of BLUPs for TCH, CCS, or Fibre target traits. β,  is a vector of fixed effects, which encompass the overall mean. The vector ε represents the random residual effects, assumed to follow a normal distribution N (0, I $\sigma_{\varepsilon }^{2}$ ), where $\sigma_{\varepsilon }^{2}$ , denote the residual variance. Here, I represent an identity matrix. The vector ***u*** corresponds to the additive genetic effects, commonly referred to as breeding values. These breeding values are assumed to follow a normal distribution $u\;∼\;N\left(0,\;G_{A}\;\sigma_{A\;}^{2}\;\right)$ , where ***G_A_*** represents the additive genomic relationship matrix and $\;\sigma_{A\;}^{2},$ is the additive genetic variance captured by SNP markers. The incidences matrix ***X*** and Z relate fixed and random effects, respectively, to observations in vector ***y*** for a specific trait.

The additive genomic relationship matrix, ${\text{G}}_{\text{A}}$ , was calculated according to (Yang et al., 2010) defined as: GA=WW'n , where W is the incidence matrix of additive genetic effects with dimensions of the number of individuals (**m** = 64,159) and number of SNPs (**n** = 10, 387). The elements of W are represented by


$${\text{w}}_{\text{ijk}\;}=\{\begin{array}{c} \frac{\left({\text{x}}_{\text{Aij}}-2{\text{p}}_{\text{i}}\right)\left({\text{x}}_{\text{Aik}}-2{\text{p}}_{\text{i}}\right)}{2{\text{p}}_{\text{i}}\left(1-{\text{p}}_{\text{i}}\right)},\;\text{j}\neq \text{k}\\ 1+\frac{{\text{x}}_{\text{Aij}}^{2}-\left(1+2{\text{p}}_{\text{i}}\right){\text{x}}_{\text{Aij}}+2{\text{p}}_{\text{i}}^{2}}{2{\text{p}}_{\text{i}}\left(1-{\text{p}}_{\text{i}}\right)},\;\text{j}=\text{k} \end{array}$$

with $p_{i}$ being the allele frequency at SNP ***i*** (where, *i* =1, 2, …, *n*), and ***x_Aij_*** is an indicator variable for additive effects that takes a value of 0, 1, and 2 if the genotype of the ***jth*** individual at SNP ***i*** is *qq*, *Qq*, or *QQ* (alleles are arbitrarily called *Q* or *q*), respectively.

For the GBLUP model (Equation. 1), the mixed model equations (MME) are:


(3) $$\left(\begin{array}{cc} X'X & X'Z\\ Z'X & Z^{\prime }Z\;+\;G_{A}^{\;\;-1}\frac{\sigma_{\varepsilon }^{2}}{\sigma_{u}^{2}} \end{array}\right)\left(\begin{array}{c} \hat{\beta }\\ \hat{u} \end{array}\right)=\left(\begin{array}{c} X'y\\ Z'y \end{array}\right)$$

(Henderson, 1984)

The solution of MME (Equation. 3) would be breeding values of clones, GEBVs. For each specific cross, a total of 50 progenies were generated. The average of the top 10% performing progenies was calculated to determine the expected breeding value associated with that cross.

In the extended-GBLUP model (Equation. 2), ***d*** and ***t*** are the random vectors of dominance deviation and additive-additive interaction deviation effects. Dominance deviation effects ***d*** were distributed as $d\;∼\;N\left(0,\;G_{D}\;\sigma_{D}^{2}\;\right)$ , where the genomic relationship matrix for dominance effects was built on genome-wide markers defined by Zhu et al. (2015) as: ${\text{G}}_{\text{D}}=\;\frac{\text{H}\;{\text{H}}^{\prime }}{\text{n}}$ , where H is the incidence matrix of dominance marker covariate matrix with dimensions of the number of individuals (***m***) and number of SNPs (***n***). The elements of H are represented by ${\text{h}}_{\text{ij}\;}=\frac{\left({\text{x}}_{\text{Dij}}-2{\text{p}}_{i}^{2}\right)}{2{\text{p}}_{\text{i}}\left(1-{\text{p}}_{\text{i}}\right)}$ , with ${\text{p}}_{\text{i}}$ being the allele frequency at SNP ***i*** (*i* =1, 2,…, *n*), and ***x_Dij_*** is an indicator variable for dominance effects that takes a value of **0, 2*pi*** and (**4*pi* − 2**) if the ***jth*** individual's genotype at SNP ***i*** is *qq*, *Qq*, or *QQ*, respectively. This parameterisation of dominance effects ensures orthogonality with additive effects. The additive-additive epistatic deviation effects were assumed to be normally distributed as $t\;∼\;N\left(0,\;G_{AA}\;\sigma_{AA}^{2}\right)$ . The additive-additive epistatic relationship matrix was represented by ***G_AA_***, calculated using the methodology by Vitezica et al. (2017). As a result, the additive-additive genomic relatedness matrix is defined as: $\frac{{\text{G}}_{\text{A}}⊙{\text{G}}_{\text{A}}}{\text{tr}\left({\text{G}}_{\text{A}}⊙{\text{G}}_{\text{A}}\right)/\text{m}}$ with ***G_A_** ⊙**G_A_*** the Hadamard product (i.e., coefficient-wise matrix product) of the additive GRM with itself, ***m*** being the number of individuals, and ***tr*** denotes the trace of the matrix (in this case, the trace of the ${\text{G}}_{\text{A}}⊙{\text{G}}_{\text{A}}$ matrix). The corresponding additive-additive interaction variance was represented by $\sigma_{AA}^{2}$ , captured using the genome-wide SNP markers. The standardisation by the average of the diagonal elements guarantees that the mean of the diagonal elements of $G_{AA}$ is nearly one as it is for ***G_A_*** and ${\text{G}}_{\text{D}}$ resulting in genetic variances estimates on the same scale as the residual variance (Vitezica et al., 2017). For each clone, the genome-wide heterozygosity was incorporated in the extended-GBLUP model as a covariate. To compute individual regression coefficients on average heterozygosity (represented by ***b*** in Equation. 2), we used an approach detailed in(Yadav et al., 2021b). We calculated the average genome-wide heterozygosity ( $Het_{k})$ for each individual clone. This value was obtained by summing up the contributions of the heterozygosity at each SNP marker, and then dividing it by the sum of twice the product of the reference allele and the alternate allele frequency at each locus.


$$Het_{k}=\frac{\sum_{i=1}^{n}h_{ki}}{\sum_{i=1}^{n}2p_{i}q_{i}}$$

Where *Het_k_* is the average genome-wide heterozygosity for an individual clone k averaged across all SNP markers and $h_{ki}$ refers to the corresponding element of dominance incidence matrix ***H*** for clone k at the ***ith*** SNP, while ${\text{p}}_{\text{i}}$ and ***q_i_*** represents the frequencies of the reference and the alternate allele at the ***ith*** SNP. The extended-GBLUP model represented by Equation. 2 is theoretically orthogonal to the additive and dominant genetic components. By orthogonality, we imply that there is no covariance between the genetic components; for example, estimates of additive genetic effects remain unbiased even when other genetic components’ effects are present in the model. The additive and dominance GRMs were computed using the GCTA software v.1.93.0b standard algorithm (Yang et al., 2011). The additive-additive GRM was computed from the additive GRM using R v.3.6.2, R-script adopted by Hivert et al. (2021). GRM computation on reasonably large data sets necessitates a substantial amount of memory. GCTA software accelerates the process by building the GRM by block, and each block is allocated to a separate thread to reduce the computational load. The GRM was constructed as a series of 8 blocks in our implementation. The different variance components of the multiple-GRM models were estimated using Linux-based software MTG2_v2.17 (Lee and Van Der Werf, 2016). MTG2 fitted models with the “direct average information” algorithm using REML for variance component estimates.

The extended-GBLUP model (Equation. 2) using Equation 4 to calculate BLUP solutions within a mixed model framework, considering three random effects.


(4) $$\left(\begin{array}{cccc} X'X & X'Z & X'Z & X'Z\\ Z'X & Z^{\prime }Z+{\text{G}}_{\text{A}}^{\;\;-1}\frac{\sigma_{\varepsilon }^{2}}{\sigma_{u}^{2}} & Z'Z & Z'Z\\ Z'X & Z'Z & Z^{\prime }Z+{\text{G}}_{\text{D}}^{\;\;-1}\frac{\sigma_{\varepsilon }^{2}}{\sigma_{d}^{2}} & Z'Z\\ Z'X & Z'Z & Z'Z & Z^{\prime }Z+{\text{G}}_{\text{AA}}^{\;\;-1}\frac{\sigma_{\varepsilon }^{2}}{\sigma_{t}^{2}} \end{array}\right)\;\left(\begin{array}{c} \hat{\beta }\\ \hat{u}\\ \hat{d}\\ \hat{t} \end{array}\right)=\left(\begin{array}{c} X'y\\ Z'y\\ Z'y\\ Z'y \end{array}\right)$$

The solution obtained from Equation 4 encompasses breeding values, dominance deviations, and additive-additive interaction deviation effects. To determine the clonal value (*ɡ*), the predicted random (breeding value, dominance deviations, additive-additive interaction deviation) effects, and the genome-wide heterozygosity effects are summed together. In the context of a specific cross, the expected clonal value is derived by averaging the top 10% performing progenies out of the total of 50 progenies generated. This approach was adopted based on the understanding that only a small percentage of the generated clones would be selected and further advanced for variety development. Breeders can identify and prioritise the most promising candidates for further evaluation and selection by focusing on the top-performing individuals within the progeny set.

Likelihood ratio tests (LRT) were employed to assess the goodness of fit for the nested GBLUP models. The LRT involved comparing the test statistic, which was calculated as two times the difference between the maximum log-likelihood of the extended GBLUP model and the maximum log-likelihood of the standard GBLUP model. In this comparison, the extended GBLUP model was considered the complete model.


Test statistic=2[max(logL(extended GBLUP))−max(logL(standard GBLUP)]

A significance threshold of **p< 0.05** was used to assess whether introducing the heterozygosity as a covariate resulted in a significant improvement of the model’s fit. If the ***Test statistic*** > $X_{\left(1\right)}^{2}$ at **p< 0.05**, the model was considered statistically significant. This approach allowed for evaluating model fit and determining the most appropriate model based on statistical significance.

#### 2.2.3 Inbreeding coefficients

The inbreeding coefficients of the progenies (Equation 5) were estimated by the diagonal element of the additive relationship matrix, $G_{A}$ , which represents the genomic relationship of an individual with itself relative to an arbitrary base population (Yang et al., 2010):


(5) $$\text{F}=\;\frac{1}{\text{n}}\;\sum_{\text{i}=1}^{\text{n}}\frac{{\text{x}}_{i}^{2}-\left(1+2{\text{p}}_{\text{i}}\right){\text{x}}_{\text{i}}+2{\text{p}}_{i}^{2}}{2{\text{p}}_{\text{i}}\left(1-{\text{p}}_{\text{i}}\right)}$$

where ***n*** is the total number of SNPs, ${\text{p}}_{\text{i}}$ , denote the allele frequency at SNP ***i*** and ${\text{x}}_{\text{i}}$ , is an indicator variable for additive genetic effects. The variable ***x_i_***, is coded as 0, 1, and 2, corresponding to the *qq*, *Qq*, and *QQ* genotypes. The coefficient F provides an unbiased estimate of the inbreeding coefficient.

The mean inbreeding of the progenies resulting from the selected sets of mating pairs was calculated to assess the influence of mate-allocation strategies on inbreeding. This mean inbreeding value was computed and averaged over all repetitions, allowing for an evaluation of the impact of mate-allocation strategies on inbreeding levels.

### 2.3 Mate-allocation

This study compared two mate-allocation strategies for selecting crossing pairs. In the first strategy, the 50 best crosses were chosen from all possible mating pairs (1,225) based on their additive value (*û*). The goal was to optimise the additive genetic gain in the next generation. In the second strategy, the best 50 crossing pairs were selected based on the predicted clonal performance ( $\hat{ɡ}$ ) to maximise the total genetic values of resulting clones.

To determine the optimal set of crosses, an integer linear programming (ILP) approach was employed using the R-lpSolve package (http://lpsolve.sourceforge.net/5.5/) (Berkelaar et al., 2015). ILP is a mathematical optimisation technique that solves static optimisation problems subject to linear equality and inequality constraints. In our case, the assumption was made that one crossing is independent of the value of the other crossing (Jansen and Wilton, 1985). Practical limitations were considered in the ILP formulation, such as the maximum number of crosses for each male and female parent. Specifically, each male parent could cross with a maximum of four female parents and vice versa. The ILP solved for binary variables ( $x_{ij})$ , where a value of 0 indicated that a particular cross between *i* ^th^ male and *j* ^th^ female was not chosen, while a value of 1 represeneted a selected crossing pair.

A total of 50 crosses were ultimately selected based on the ILP optimisation process. The expected additive gain Δu(=*mean*(*û*_50_)-*mean*(*û*_1225_)) was calculated as the difference between the mean breeding value of the selected 50 mating pairs and the mean breeding value of all possible crossing pairs (1,225). Similarly, the expected total genetic superiority $\Delta \text{g }\left(=mean({\hat{ɡ}}_{50}\right)-mean\left({\hat{ɡ}}_{1225}\right))$ was determined as the difference between the mean clonal value of the selected 50 crosses and the mean clonal value of all possible crossing pairs. The results presented in this study are based on the average of ten repetitions of each simulation, providing reliable insights into the performance of the mate-allocation strategies.

## 3 Results

### 3.1 Variance components and heritabilities

The estimates of variance components, heritabilities, and the maximum log-likelihood ratio values obtained from the two described models (Equations 1 and 2) are shown in **Table 1** for the three traits examined. TCH exhibited the lowest narrow-sense (*h^2^* = 0.18) heritability estimates using the extended-GBLUP model, while CCS and Fibre had relatively high heritability estimates (CCS: *h^2^* = 0.4, Fibre: *h^2^* = 0.5). Notably, estimates of additive genetic variance differed significantly between the GBLUP and the extended-GBLUP models, particularly for TCH. For additive variance, the standard errors for TCH using the GBLUP model were higher compared to the extended model, whereas the standard errors for the other two traits remained the same regardless of the model.

**Table 1:** Estimates of additive and non-additive (dominance and epistatic) variance components, narrow-sense heritability, dominance, additive-additive interaction ratio, log-likelihood ratio (LKH), and heterozygosity effects (*b*) for TCH, CCS and Fibre for 2,909 clones.

Trait	Model	$\sigma_{\varepsilon }^{2}$	$\sigma_{A}^{2}$	$\sigma_{D}^{2}$	$\sigma_{E}^{2}$	*h^2^*	$d^{2}$	*E^2^*	LKH	Heterozygosity effects (*b*)
TCH	GBLUP	68.51 (2.26)	24.91 (3.33)			0.27 (0.03)			7874.67^a^	
	e-GBLUP	46.10 (3.94)	15.82 (2.9)	3.08 (2.05)	22.94 (4.74)	0.18 (0.03)	0.04 (0.02)	0.26 (0.05)	7809.28^b^	92.84* (11.08)
CCS	GBLUP	0.25 (0.009)	0.24 (0.02)			0.49 (0.03)			15.05^a^	
	e-GBLUP	0.21 (0.02)	0.19 (0.02)	~ 0 (NA)	0.07 (0.02)	0.40 (0.03)	~ 0 (NA)	0.15 (0.04)	25.83^b^	0.29^ns^ (0.19)
Fibre	GBLUP	0.81 (0.03)	0.90 (0.08)			0.53 (0.03)			1729.24^a^	
	e-GBLUP	0.68 (0.06)	0.85 (0.08)	~ 0 (NA)	0.17 (0.07)	0.5 (0.03)	~ 0 (NA)	0.1 (0.04)	1725.26^b^	-0.05^ns^ (1.22)

$\sigma_{A}^{2}$ = additive genetic variance;= dominance genetic variance; $\sigma_{E}^{2}$ = epistatic (additive-additive) genetic variance; $\sigma_{\varepsilon }^{2}$ = residual variance; standard error (SE) in parentheses. LKH, Log Likelihood Ratio. (a-b) models without a common superscript are significantly different at p< 0.05.

Model GBLUP, classical additive model; Model e-GBLUP, extended-GBLUP includes non-additive effects with average heterozygosity of clones with additive effects. TCH, Tonnes of cane per hectare; CCS (%), Commercial cane sugar; Fibre (%), Fibre content. (b), a change in phenotypic mean per 1% increase in heterozygosity for a particular trait. *,means significant effects; ns reflect non-significant effects.

The narrow-sense heritability (*h^2^*) estimates for CCS and Fibre were comparable in both GBLUP and extended-GBLUP models. When additive, dominance, epistatic genetic effects, and heterozygosity effects were simultaneously included in the model, the estimates of dominance variance for CCS and Fibre were close to zero. In contrast, the dominance effects explained nearly 4% of the phenotypic variance for TCH (**Table 1**). Additionally, TCH exhibited the highest ratio of dominance genetic variation to additive variation (0.19), with additive-additive interaction contributing to around 55% of overall genetic variation. In contrast, additive variance explained about 38% of genetic variance.

**Figure 3:**
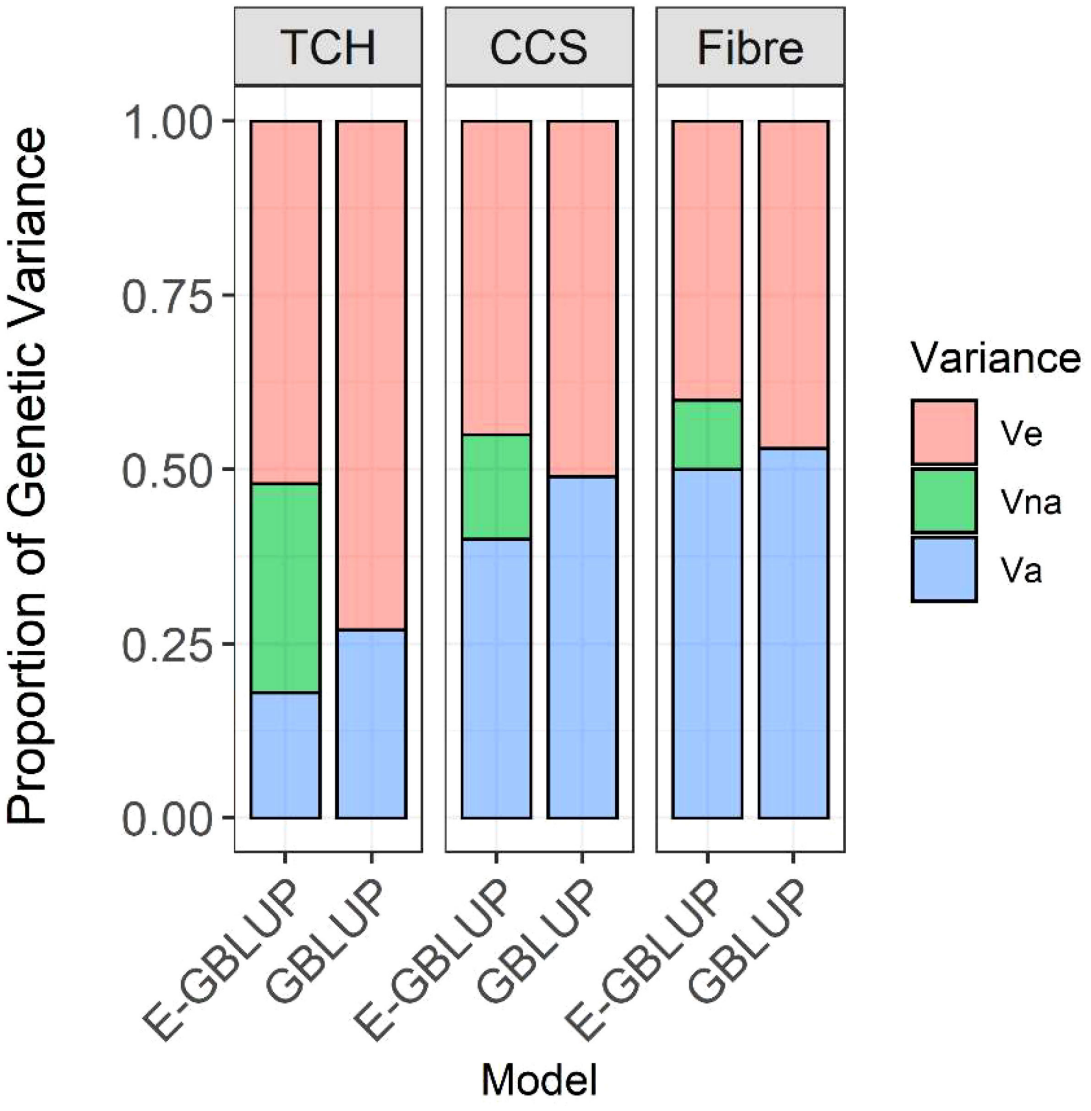
Decomposition of genetic variance into additive and non-additive and residual variance; V_a_, additive genetic variance; V_na_, non-additive genetic variance (including dominance and additive-additive interaction variance components); V_e_, error variance; Model GBLUP, traditional additive model; E-GBLUP, extended-GBLUP model by integrating additive, dominance, epistatic and heterozygosity effects.

Incorporating non-additive (dominance and additive-additive interaction) genetic effects and heterozygosity effects resulted in a significant reduction in residual variance for all traits compared to the additive model (**Table 1**, **Figure 3**). This indicates that a portion of the non-additive genetic variation was captured within the residual variance in the GBLUP model. The additive-additive interaction variation accounted for about 27% of the total genetic variance in CCS, while for Fibre, the epistatic variance was the lowest, contributing to only 17% of the overall genetic variance. Based on the log-likelihood, the extended-GBLUP provided a better fit to the data than the regular GBLUP model, regardless of the traits under consideration.

### 3.2 Effect of heterozygosity

The average heterozygosity per clone was calculated based on the heterozygosity across markers. The regression coefficient for heterozygosity in relation to TCH (92.84 ± 11.08) was found to be significant, suggesting that an increase in genome-wide heterozygosity is associated with an increase in average cane yield. However, for CCS and Fibre, the standard error of the regression coefficient was considerably larger compared to the heterozygosity estimates, and the difference was not statistically significant (**Table 1**).

### 3.3 Mate-allocation strategies

The total variation in predicted breeding $\hat{u}$ (and clonal $\hat{ɡ}$ ) value for all potential crossing pairs (n=1,225), the 50 best crosses selected through ILP, and the top decile of best crosses are depicted in **Supplementary Figure S1** for TCH, CCS and Fibre in one simulation iteration. The mean $\hat{u}$ (or $\hat{ɡ}$ ) for all potential crossing pairs (the baseline for our comparisons, n=1,225) was 2.02 ± 0.006 (4.80 ± 0.02) tonnes/ha for TCH, 0.24 ± 0.0008 (0.31 ± 0.0009) measured in % for CCS, and 0.46 ± 0.002 (0.61 ± 0.002) in% for Fibre across ten iterations (**Table 2**). Compared to the additive model (GBLUP), the average expected progeny value of selected matings based on the model that incorporated non-additive genetic effects (e-GBLUP) showed improvements of 57%, 12%, and 16% for TCH, CCS, and fibre, respectively, compared to an additive model (GBLUP) (**Table 2**).

**Table 2:** Average expected progeny value (EPV_avg_) and genomic measures of average inbreeding of progenies (PIB_avg_) of selected crosses (50) across ten iterations of simulation for TCH, CCS, and fibre with standard deviations in parentheses.

Mate-allocation	TCH (tonnes/ha)		CCS (%)		Fibre (%)	
Selection of crossing pairs	EPV_avg_	PIB_avg_	EPV_avg_	PIB_avg_	EPV_avg_	PIB_avg_
GEBV^1^	5.29 (0.06)	0.04 (0.01)	0.50 (0.003)	0.02 (0.01)	1.16 (0.01)	0.02 (0.009)
**GPCP^2^**	8.28 (0.07)	-0.046 (0.007)	0.56 (0.005)	0.03 (0.01)	1.34 (0.02)	0.05 (0.007)

^1^Mate-allocation strategy, where crossing pairs would be selected based on GEBVs; ^2^Mate-allocation strategy, where crossing pairs would be selected based on GPCPs; GEBV, genomic estimated breeding value; GPCP, genomic prediction of clonal performance exploiting non-additive effects; TCH, tonnes cane per hectare; CCS, commercial cane sugar; Fibre, Fibre content. The standard deviation in parentheses reflects the variation across ten simulation repetitions.

**Figure 4:**
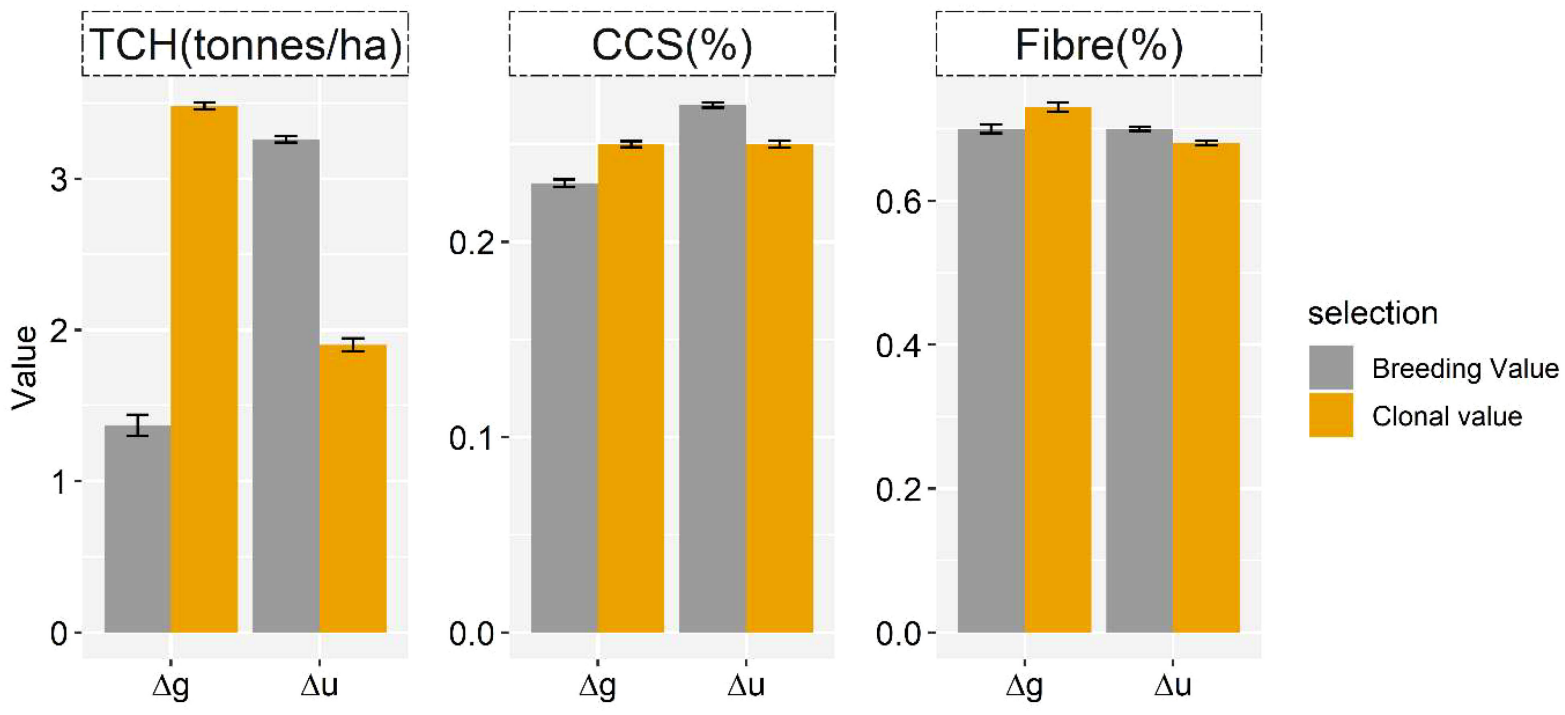
The x-axis represents the expected gain in terms of total clonal performance (Δ*g*), reflecting variety performance and total additive (Δ*u*) gain, pertaining to parental selection obtained from 50 selected crosses using a linear programming optimisation algorithm. Δu = ***û*_50_** − ***û*_1225_**; Δg = ***ĝ*_50_** − ***ĝ*_1225_**; where ***û*_1225_** (or ***ĝ*_1225_**) is the mean breeding (or clonal) value of total potential crossing pairs, and ***û*_50_** (or ***ĝ*_50_**) is the mean breeding (or clonal) value of selected crossing pairs using the integer linear programming optimisation technique. The error bar shows the standard error of the mean for ten repetitions of the simulation.

The average genomic inbreeding coefficient of progenies for TCH indicated that the selected progenies based on the extended-GBLUP model with mate allocation had lower estimates of inbreeding than the additive model (**Table 2**). The negative genomic inbreeding estimates reflect that the selected clones in crosses were more heterozygous (less inbred) than the average. The difference between the mean $\hat{u}\;(or\;\hat{ɡ}$ ) of the selected crosses, and the mean of all potential matings was referred to as the expected additive genetic gain (Δu) and expected total genetic superiority (Δg), respectively. **Figure 4** illustrates the additive genetic gain (Δu) and total clonal superiority (Δg) obtained with the selected matings for each mating strategy.

The expected total genetic superiority of the progeny was higher for all traits when matings were chosen based on clonal performance (GPCP) rather than breeding value (GEBV), providing the offspring with an advantage for TCH, CCS, and Fibre. However, a significant decrease in additive genetic gain was found using the same selection strategy, notably for TCH. There were no major differences in the additive (Δu) and expected genetic (Δg) gain for CCS and Fibre in a specific mate-allocation strategy. The rankings of crossing pairs varied significantly depending on whether the selection was based on GEBVs or GPCPs. For example, we observed that out of the selected 50 crossing pairs, there was an overlap of only approximately six pairs between the mate-allocation strategies for TCH. This limited overlap can be attributed to the higher epistatic effects associated with this trait. This indicates that different parents were selected to optimise the total additive (or clonal) value.

## 4 Discussion

Our simulation study demonstrates that mate-allocation strategies that account for non-additive genetic effects can improve progeny performance in the next generation. Considering non-additive genetic effects in mating decisions would likely lead to breeding higher-performing varieties. We observed a substantial improvement in the average expected progeny value of selected crossing pairs, with increases of 57%, 12%, and 16% for TCH, CCS, and Fibre, respectively, when non-additive and heterozygosity effects were exploited. These results aligned with other clonal crops and outbred species (Aliloo et al., 2017; Gonzalez-Dieguez et al., 2019; Werner et al., 2023; Wolfe et al., 2021). For instance, Toro and Varona (2010) reported a 6% to 22% improvement in offspring performance through mate-allocation considering dominance genetic effects in a simulation study. In contrast, Ertl et al. (2014) demonstrated an estimated average genetic superiority increase of a 14.8% increase in milk yield and a 27.8% increase in protein yield using mate-allocation techniques in cattle breeding using empirical data. Furthermore, Resende (2014) achieved up to a 113% improvement in phenotypic benchmarks by exploiting dominance effects in loblolly pine (*Pinus taeda* L.) tree breeding.

As sugarcane breeding programs progress, inbreeding and a reduction in genetic diversity pose risks of inbreeding depression. It is crucial to carefully select and allocate mates to strike a balance between genetic gain and the negative consequences of increased inbreeding. Lin et al. (2017) also suggested implementing inbreeding controls during mate-allocation when employing GS in outbred plants. In this study, selecting crossing pairs while accounting for genome-wide heterozygosity and non-additive effects considerably reduced progeny inbreeding, especially for TCH, which aligns with previous research (Aliloo et al., 2017; Gonzalez-Dieguez et al., 2019).

A range of mate-selection indices, such as the optimal contribution (Wray and Goddard, 1994; Meuwissen, 1997), superior progeny value (Zhong and Jannink, 2007), and its extensions, such as optimal population value (Goiffon et al., 2017) and usefulness criterion (Lehermeier et al., 2017; Wolfe et al., 2021), have been developed to balance gains from selection with average inbreeding and co-ancestry. In addition, genetic distance has also been used to evaluate parental genetic differences and, as a result, to predict heterosis and select parents for a cross. However, the relationship between heterosis and genetic distance remains unclear due to inconsistent findings from various studies (Cheres et al., 2000; Yingbin et al., 2019; Liu et al., 2022). However, clonally propagated crops, including sugarcane, are highly heterozygous polyploids, where virtually all gametes produced by any parental genotype are distinct, and all F_1_ descendants are unique (Wei et al., 2021). Leveraging the overall genetic effects, both additive and non-additive can be employed to develop new varieties. Cross-prediction within a population is critical for population improvement in clonally propagated crops. Some recent crop studies also advocated strategies for selecting parents in artificial crosses based on the genomic prediction of cross performance by leveraging the non-additive effects in cereal, e.g. wheat (Lado et al., 2017) and clonal crops, e.g. strawberry and cassava (Werner et al., 2023; Wolfe et al., 2021). Thus, mate-allocation is the most straightforward approach to harness the benefits of non-additive effects among all the strategies.

Our simulation results revealed a clear relationship between improved progeny performance in the next generation and genome-wide heterozygosity, particularly for TCH, where significant heterozygosity effects were observed. The extend-GBLUP model, when heterozygosity is not explicitly accounted for, may lead to an overestimation of dominance variance (Iversen et al., 2019). The same observation has been made in our previous study (Yadav et al., 2021b), which used the same dataset as this study; we observed a considerable reduction in dominance variance for TCH after including genome-wide heterozygosity per clone in addition to a random dominance term.

Our results demonstrate that selecting crossing pairs based on cross-performance exploiting non-additive effects and heterozygosity effects yields higher genetic gain (clone performance) than selection based solely on breeding value, which is consistent with previous studies (Ertl et al., 2014; Resende, 2014; Toro and Varona, 2010; Aliloo et al., 2017; Fernandez et al., 2021). Among the traits studied, TCH exhibited the greatest increase in overall genetic gain, as non-additive genetic effects accounted for nearly two-thirds of the genetic variation. However, our results contrast sharply with mate-allocation techniques used in cattle and pig breeding schemes, where the inclusion of non-additive genetic effects to exploit heterosis resulted in a higher predicted total genetic superiority while only minimally reducing expected additive gain (Ertl et al., 2014; Gonzalez-Dieguez et al., 2019). One of the key explanations could be the substantial magnitude of dominance and epistatic effects in sugarcane compared to these studies, resulting in the selection of different sets of parents to optimise clonal and additive values in the crossing pairs. ILP was utilised in this study to facilitate the decision-making process in determining the optimal combination of these crossing pairs, taking into account the practical limitations of breeding programs, such as restricting the number of parents that a parent may cross with. This optimisation technique is widely applied in mate allocation research to improve decision-making (Resende, 2014; Aliloo et al., 2017; Gonzalez-Dieguez et al., 2019).

Under the assumption of Hardy-Weinberg equilibrium, the total genetic variance was partitioned into additive, dominance, and epistatic variance. Although the model is theoretically orthogonal, our results demonstrate a reduction in additive variance for all traits when non-additive genetic effects are included in the model. These results are consistent with those obtained when applying the natural and orthogonal interaction (NOIA) model to the same population of clones where the Hardy-Weinberg equilibrium condition was relaxed (Yadav et al., 2021b). High linkage disequilibrium in modern elite sugarcane clones might explain some confounding effects of additive and non-additive genetic effects (Jannoo et al., 1999; Raboin et al., 2008). Most significantly, a simple diploid model is unlikely to reflect all of the sugarcane’s genomic and genetic complexity. These confounding effects pose challenges in pinpointing the specific genetic contributions to observed phenotypic variation, thus hindering the interpretation of the relative importance of additive and non-additive effects on the expression of sugarcane traits. Moreover, the reduction in residual variance observed in the extended-GBLUP model indicates that residual variation contains a significant portion of the non-additive variation not captured by the traditional GBLUP model, which does not account for non-additive effects. Other studies have come up with similar findings (Aliloo et al., 2017; Gonzalez-Dieguez et al., 2019). As a result, the residual genetic variance may include high-order non-additive genetic variation that is not captured by markers and error variance.

It is important to acknowledge that mate-allocation strategies used in this study consider truncation selection and planned mating based on additive or total genetic performance. It might result in the selection of close relatives. This focus on selecting the top-performing lines offers short-term genetic progress; however, it poses a risk of limiting long-term genetic gain due to high rates of population inbreeding. To balance long- and short-term gain, the sugarcane breeding scheme can be viewed as having two interconnected goals: recurrent GS for population improvement and the development of varieties for immediate use. Recurrent GS focuses on allele substitution effects, which control and increase the frequency of favourable alleles in the population over time and can be primarily driven for larger genetic gain in the long term. On the other hand, the variety development pipeline utilises non-additive and heterozygosity effects to improve the phenotypic performance of market-ready clones, prioritising short-term goals. However, it is important to note that preselecting clones based on GEBVs might restrict the opportunity to select alternative clones that might potentially generate offspring with higher overall genetic value in specific matings. It is crucial to consider that mating, which benefits from non-additive effects, can only increase progeny performance during its implementation, and the benefits resulting from specific combining abilities cannot be accumulated over multiple generations. Therefore, exploring innovative methods for optimizing genetic contributions (e.g., optimal contribution selection) can be advantageous to balance short-term gain and preservation of long-term genetic diversity.

Our results are limited to a specific population and focus on a single trait. Consequently, the proposed approach could lead to the selection of different parental lines when targeting different traits. In practice, however, expanding the approach to multiple-trait selection is preferable. A simple extension would be to use a selection index that includes multiple traits and then consider the selection index as a new target trait for the existing single-trait approach. Another possible modification is directly implementing multi-trait genomic prediction models and evaluating selection lines using an appropriate selection index.

Furthermore, in order to approximate a high-complexity genome, we used simplified assumptions in our simulation schemes. However, genetics in auto-polyploid species is more complicated than in diploid species since more than two alleles may occur at the same locus. As a result, there are additional phases and recombination, and preferential pairing can vary. In addition, there is limited theoretical and experimental information on recombination and segregation in high-ploidy species. And determining which alleles co-occur on each homologous copy gets increasingly challenging as ploidy increases. Furthermore, a reference genome is essential for phasing genotypes in heterozygous polyploids like sugarcane. Unfortunately, the sugarcane community does not have access to the complete reference sequence, which did not allow for leverage of genome-wide phased haplotypes. Nevertheless, the pseudo-diploid markers are a rough approximation of what we used in our study because the majority of the markers in the SNP array are single/low-dose markers. Based on genotype allele count (0, 1 and 2), the software we used to simulate the progenies assumes that the input (marker) data is in the correct gametic phase.

We acknowledge the limitations of our research, particularly the simplifications inherent in using a basic diploid parametrisation and the challenges in representing sugarcane’s complex genomic and meiotic characteristics. The availability of the Affymetrix SNP array, with its considerable number of single-dose SNP markers, has facilitated our study. Still, it is important to recognise that this approach may not entirely capture the genetic complexity of sugarcane. Furthermore, the small size of our training population and the impact of environmental variations on genotypic values should be taken into account when selecting cross combinations. Therefore, conducting field trials to validate our findings would be a valuable next step. Additionally, further research is required to fully understand the benefits of mate-allocation methods in a larger context.

## 5 Conclusion

In conclusion, our simulation study demonstrates that implementing genomic mate-allocation strategies that consider non-additive genetic effects holds promise as a feasible and effective method for enhancing the performance of clonal offspring in sugarcane breeding programs. Across all traits evaluated, the inclusion of non-additive effects in mate-allocation strategies yielded favourable outcomes, resulting in a substantial 57% improvement in cane yield performance compared to strategies focused solely on additive effects. Moreover, when crossing pairs were selected to leverage non-additive and heterozygosity effects, the average inbreeding coefficient of the progeny was significantly reduced, particularly in cases where the target trait was TCH. This reduction in the inbreeding coefficient helps to preserve long-term genetic gains and maintain the overall genetic diversity within the population.

One notable advantage of mate-allocation methods that account for non-additive genetic effects and heterozygosity is their relative ease of implementation compared to other approaches described in the literature. By directly estimating the performance of progeny in the subsequent generation, these methods provide a practical and efficient means of incorporating non-additive effects into breeding operations. This becomes especially valuable when breeding operations involve time-consuming processes such as establishing segregating populations and conducting field evaluations, emphasising the importance of systematic planning in cross-selection. Although genomic mate-allocation contributes to the overall improvement of offspring performance in our simulation study, it is crucial to note that it does come at the cost of reduced expected additive genetic gain, particularly in the case of cane yield. Therefore, future breeding programs will face the interesting challenge of balancing long-term gains derived from selection on additive effects and the short-term development of high-performing varieties. We emphasize that these findings are derived from simulation experiment, and their real-world applicability should be considered in light of the simulation-based nature of this study,

## Data availability statement

The datasets presented in this article are not readily available due to confidentiality agreements. The modified marker data set and genetic map for sugarcane are made available on GitHub at this link https://github.com/SimmiSudhir/Linkage-Disequilibrium-Algorithm. Further inquiries can be directed to the corresponding author.

## Author contributions

SY: Data curation, Formal Analysis, Methodology, Software, Visualization, Writing – original draft, Writing – review & editing. ER: Supervision, Writing – review & editing. XW: Formal Analysis, Investigation, Project administration, Writing – review & editing. OP: Supervision, Writing – review & editing. VH: Software, Writing – review & editing. LH: Supervision, Writing – review & editing. FA: Project administration, Writing – review & editing. ED: Data curation, Formal Analysis, Writing – review & editing. KA: Resources, Writing – review & editing. KV-F: Supervision, Writing – review & editing. BH: Conceptualization, Funding acquisition, Investigation, Project administration, Resources, Supervision, Validation, Writing – review & editing, Methodology.

## Supplementary material

Supplementary Figure 1 (**A)** Predicted breeding value (left) and clonal value (right) of 1225 crossing pairs, best 50 crosses and top decile of best 50 crosses in one iteration of simulation for TCH; (**B)** Predicted progeny (breeding/clonal) value for CCS; (**C)** Predicted progeny value for fibre content. The “+” sign represents the mean value, and the solid line across the box represents the median.
