# Supplementary material for: Optimising clonal performance in sugarcane: leveraging non-additive effects via mate-allocation strategies
Source: Front Plant Sci. 2023 Nov 10;14:1260517. doi: 10.3389/fpls.2023.1260517 (PMC10667552; doi:10.3389/fpls.2023.1260517)
Supplement: Supplementary Figure 1 — (A) Predicted breeding value (left) and clonal value (right) of 1225 crossing pairs, best 50 crosses and top decile of best 50 crosses in one iteration of simulation for TCH; (B) Predicted progeny (breeding/clonal) value for CCS; (C) Predicted progeny value for fibre content. The “+” sign represents the mean value, and the solid line across the box represents the median. [file Image_1.pdf]

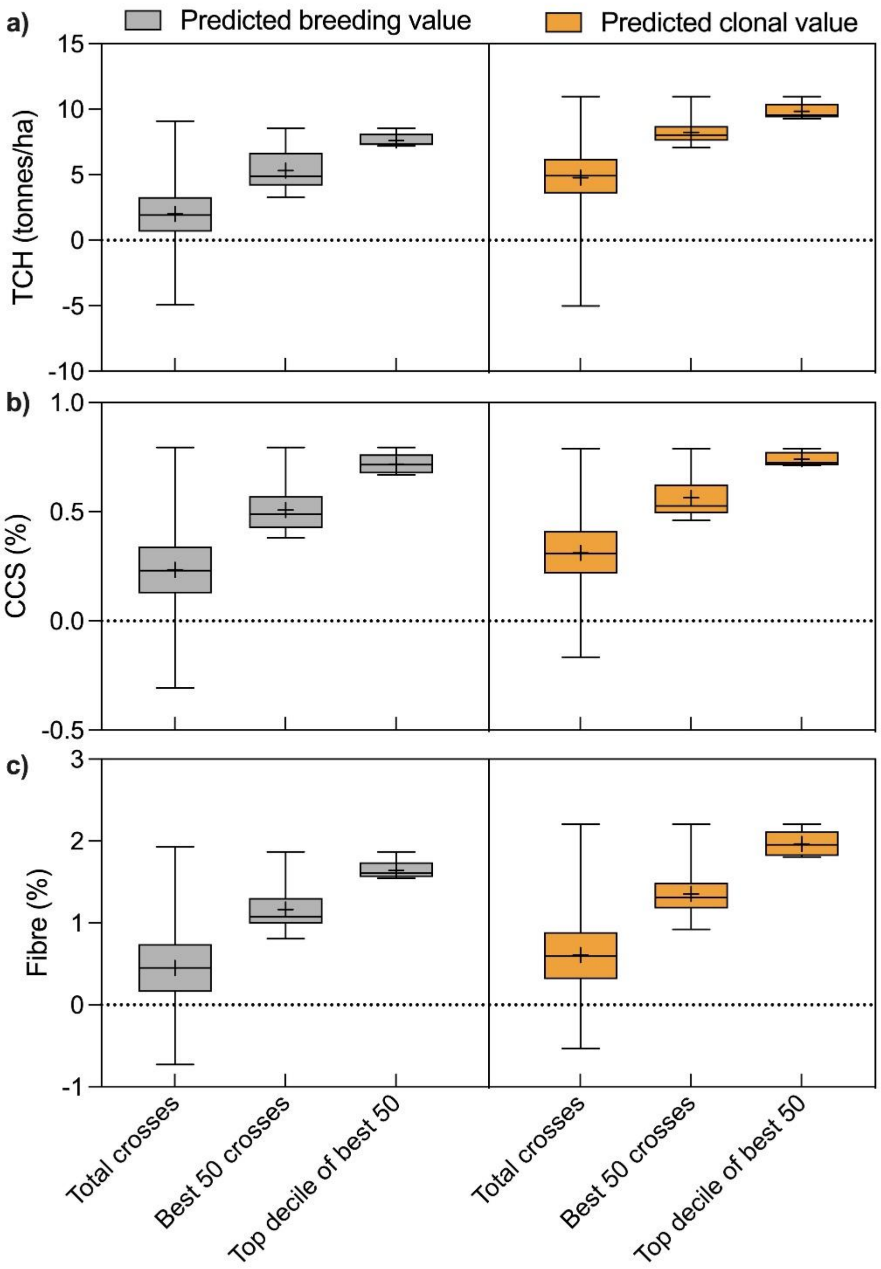

Fig.S1 a) Predicted breeding value (left) and clonal value (right) of 1225 crossing pairs, best 50 crosses and top decile of best 50 crosses in one iteration of simulation for TCH; **b)** Predicted progeny (breeding/clonal) value for CCS; **c)** Predicted progeny value for fibre content. The “+” sign represents the mean value, and the solid line across the box represents the median.
